# Supplementary material for: Inverse identification of region-specific hyperelastic material parameters for human brain tissue
Source: Biomech Model Mechanobiol. 2023 Sep 7;22(5):1729–49. doi: 10.1007/s10237-023-01739-w (PMC10511383; doi:10.1007/s10237-023-01739-w)
Supplement: Supplementary file 1 — Supplementary file1 (PDF 1334 kb) [file 10237_2023_1739_MOESM1_ESM.pdf]

Supplementary material: Inverse identification of region-specific hyperelastic material parameters for human brain tissue

Jan Hinrichsen[1], Nina Reiter[1], Lars Bräuer[2], Friedrich Paulsen[2], Stefan Kaessmair[1], and Silvia Budday*[1]

[1]*Institute of Applied Mechanics, Friedrich Alexander University Erlangen-Nürnberg, 91058 Erlangen, Germany,*
[2]*Institute of Functional and Clinical Anatomy, Friedrich Alexander University, 91054 Erlangen, Germany*

1 Parameter sets

	preconditioned ν =45				preconditioned ν =49				unconditioned ν =45				unconditioned ν =49			
	α		μ		α		μ		α		μ		α		μ	
	mean	std.	mean	std.	mean	std.	mean	std.	mean	std.	mean	std.	mean	std.	mean	std.
gov. region																
Am	-19.67	1.80	257.05	116.18	-14.16	1.60	193.34	100.33	-14.74	2.55	386.97	171.00	-10.10	2.26	293.06	133.23
BG	-18.95	4.02	252.15	99.15	-15.05	4.80	169.67	66.85	-15.90	4.51	338.67	135.90	-11.49	4.14	246.21	97.77
BS	-20.41	6.47	233.12	112.45	-19.59	8.21	118.06	43.22	-16.82	6.11	336.44	165.75	-13.60	5.99	225.08	106.82
C	-19.27	4.42	281.75	114.14	-16.72	5.05	175.21	73.39	-16.16	2.92	392.24	153.61	-12.16	2.79	285.97	110.82
CB	-23.22	5.96	177.49	81.38	-18.79	3.76	115.36	48.54	-19.49	4.03	255.38	100.99	-14.08	3.40	196.55	88.38
CC	-14.94	12.60	61.66	24.73	-13.72	13.71	37.64	18.26	-12.82	7.88	95.43	34.55	-9.11	6.90	67.82	24.40
CR	-19.70	4.73	157.09	75.55	-19.08	7.60	87.58	47.53	-16.93	6.60	218.75	115.76	-13.55	6.94	146.54	77.74
Hi	-17.37	2.75	184.21	81.38	-13.09	3.15	129.96	47.59	-13.25	1.58	292.58	147.86	-9.23	1.33	218.74	112.20
M	-18.68	4.99	215.70	74.61	-15.15	5.96	137.67	37.76	-15.11	4.97	314.92	110.47	-10.89	4.19	227.69	74.32

Table S1: Material parameters averaged over governing regions.

	preconditioned ν =45				preconditioned ν =49				unconditioned ν =45				unconditioned ν =49			
	α		μ		α		μ		α		μ		α		μ	
	mean	std.	mean	std.	mean	std.	mean	std.	mean	std.	mean	std.	mean	std.	mean	std.
region																
Am	-19.67	1.80	257.05	116.18	-14.16	1.60	193.34	100.33	-14.74	2.55	386.97	171.00	-10.10	2.26	293.06	133.23
CC	-14.94	12.60	61.66	24.73	-13.72	13.71	37.64	18.26	-12.82	7.88	95.43	34.55	-9.11	6.90	67.82	24.40
CI	-21.20	-	205.14	-	-19.49	-	116.44	-	-18.37	-	261.34	-	-13.99	-	188.13	-
CR	-19.83	2.96	155.69	67.08	-19.28	5.99	83.33	27.13	-18.65	2.02	209.72	97.64	-15.78	3.89	136.21	57.59
FC	-19.63	0.97	249.69	82.67	-15.90	1.90	168.72	53.82	-15.29	1.73	361.23	127.01	-11.08	1.75	271.89	96.14
Hi	-17.37	2.75	184.21	81.38	-13.09	3.15	129.96	47.59	-13.25	1.58	292.58	147.86	-9.23	1.33	218.74	112.20
M	-18.72	4.87	230.60	99.27	-16.35	6.36	135.61	42.66	-15.07	4.16	338.29	137.77	-11.30	4.13	238.30	91.76
MC	-20.19	1.64	318.98	126.18	-17.99	3.06	195.50	84.99	-16.95	2.06	434.35	168.54	-12.94	2.19	313.65	119.22
Me	-18.90	7.63	228.86	106.41	-17.96	9.30	120.53	41.93	-16.11	7.20	323.46	161.20	-13.15	6.92	216.22	107.02
NC	-15.82	5.85	198.57	99.88	-11.39	5.73	143.46	74.60	-12.58	6.10	277.58	138.29	-8.30	4.94	203.23	96.48
P	-23.23	1.31	241.04	131.47	-22.62	4.91	113.46	48.61	-18.12	3.38	360.56	184.33	-14.43	4.09	241.52	112.86
Pa	-21.16	2.14	242.06	76.14	-17.94	3.75	154.22	60.56	-18.19	3.07	332.32	137.93	-13.67	3.21	240.96	105.92
Pu	-19.24	1.76	305.10	100.76	-15.09	2.90	206.07	55.33	-16.27	2.61	393.89	121.48	-11.86	2.75	285.82	82.46
TL	-8.37	13.00	123.68	23.29	-4.38	11.03	89.51	23.47	-9.18	3.07	202.19	75.27	-5.95	1.97	150.13	61.67
Th	-18.66	5.27	205.38	53.76	-14.32	5.78	139.10	35.73	-15.13	5.63	298.74	89.50	-10.60	4.38	220.34	62.52
VC	-20.81	0.58	299.02	67.23	-19.10	1.09	174.95	47.48	-17.64	1.70	421.88	107.24	-13.62	1.80	306.03	90.17
WM	-19.62	5.66	158.00	82.06	-18.95	8.62	90.34	57.54	-15.81	8.21	224.64	127.98	-12.10	8.11	153.27	89.07
cN	-22.66	4.42	123.64	38.58	-17.43	2.83	89.13	39.37	-17.50	2.53	203.00	84.30	-12.70	2.90	155.49	74.71
cWM	-23.57	7.03	211.15	84.48	-19.65	4.18	131.76	48.53	-20.74	4.43	288.12	101.18	-14.94	3.57	222.22	90.80

Table S2: Material parameters averaged over anatomical regions.

2 Normality

ν	precond	RMSE		α		μ	
		W	p	W	p	W	p
0.45	preconditioned	0.861346	7.426005e-12	7.35e-01	9.06e-17	9.61e-01	5.68e-05
0.45	unconditioned	0.843182	1.023565e-12	8.29e-01	2.53e-13	9.57e-01	2.38e-05
0.49	preconditioned	0.821075	1.123478e-13	9.29e-01	8.81e-08	9.46e-01	2.15e-06
0.49	unconditioned	0.844200	1.138903e-12	8.90e-01	2.43e-10	9.54e-01	1.24e-05

Table S3: Results for the Shapiro Wilk test for the material parameters μ and α yielding the probability that the observations stem from a normal distribution.

3 Regional dependency

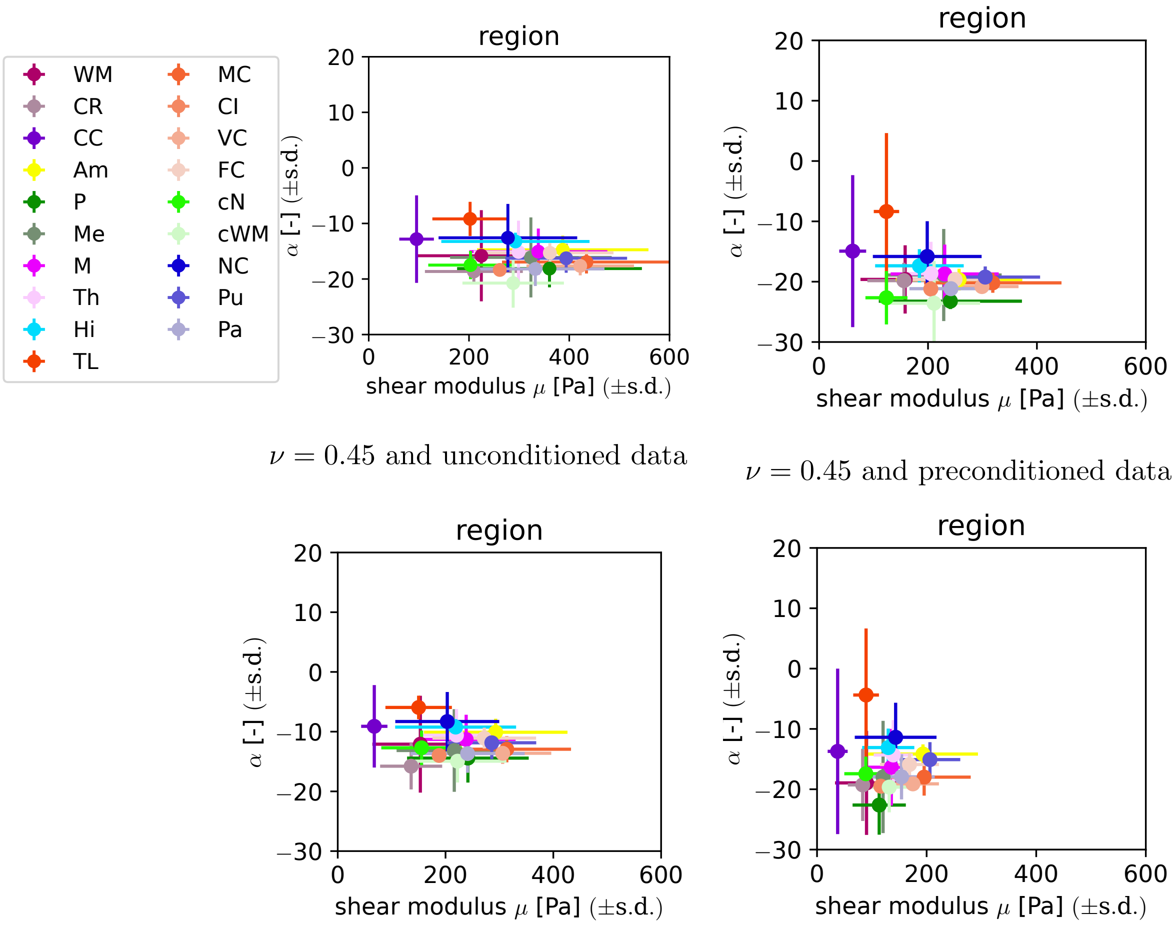

$\nu = 0.45$ and unconditioned data

$\nu = 0.45$ and preconditioned data

$\nu = 0.49$ and unconditioned data

$\nu = 0.49$ and preconditioned data

Figure S1: Material parameter distribution for different anatomical regions.

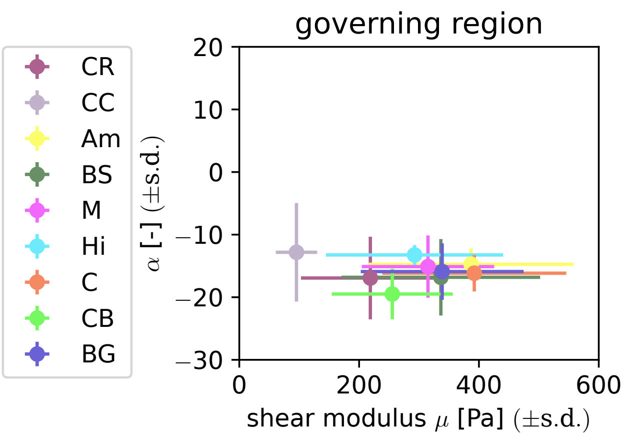

$\nu = 0.45$ and unconditioned data

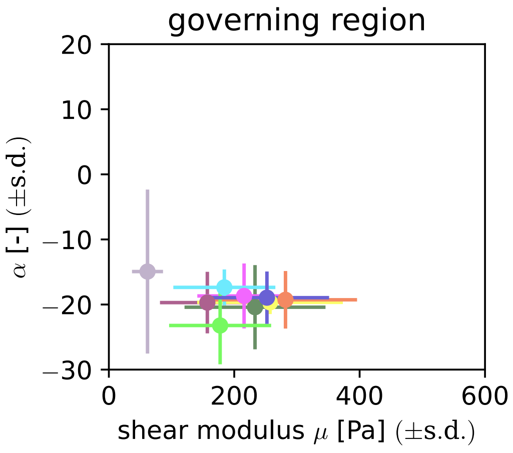

$\nu = 0.45$ and preconditioned data

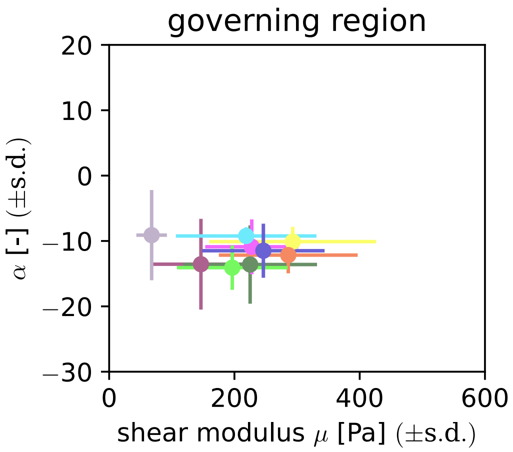

$\nu = 0.49$ and unconditioned data

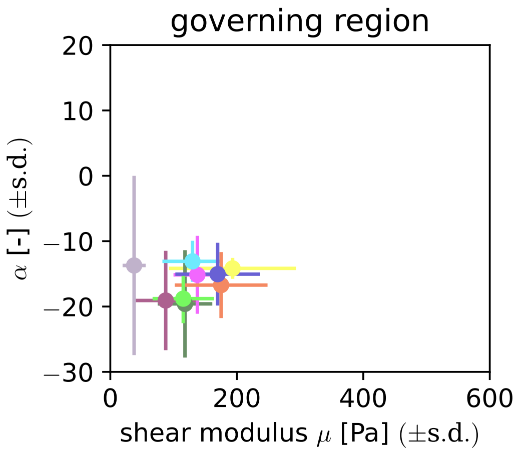

$\nu = 0.49$ and preconditioned data

Figure S2: Material parameter distribution for the defined governing regions.

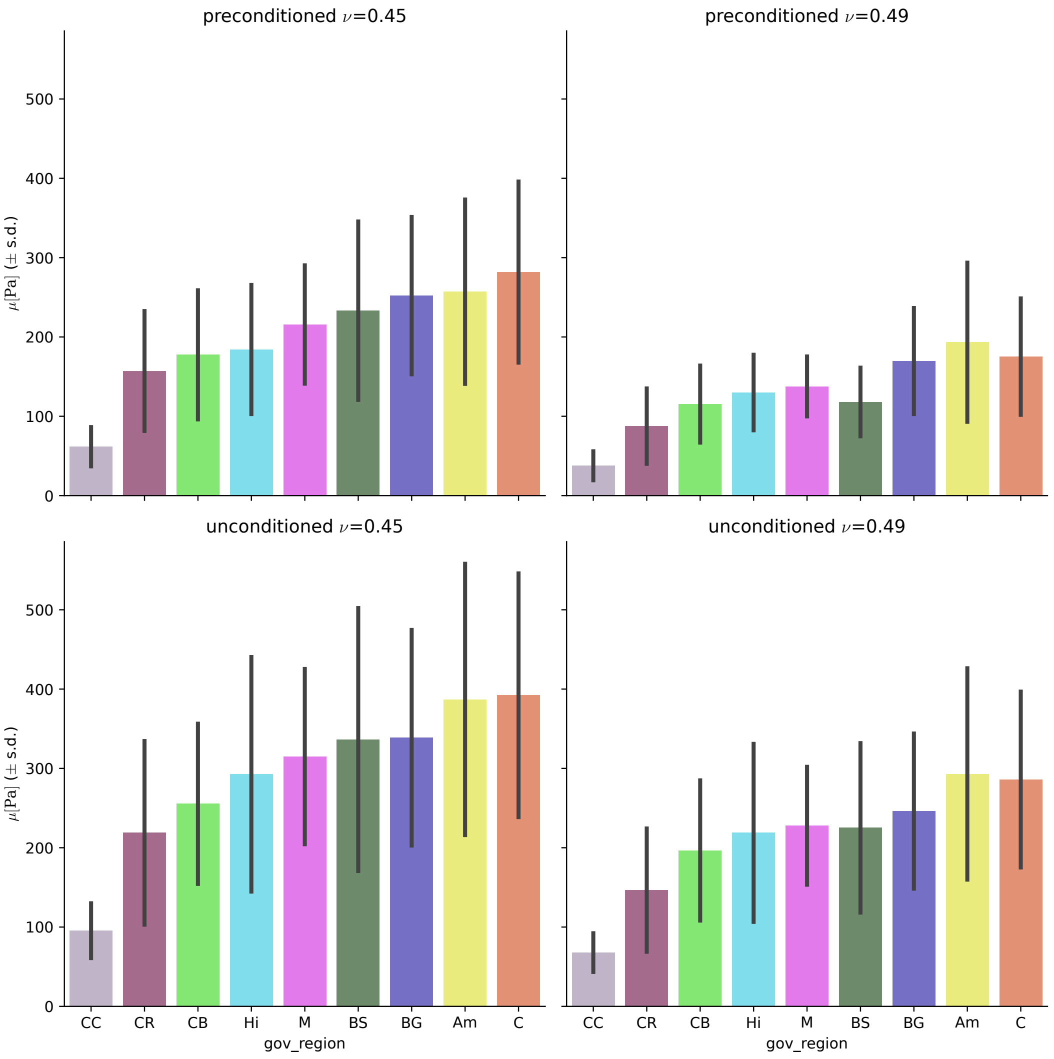

Figure S3: Resulting values for the shear modulus μ, averaged over the governing regions.

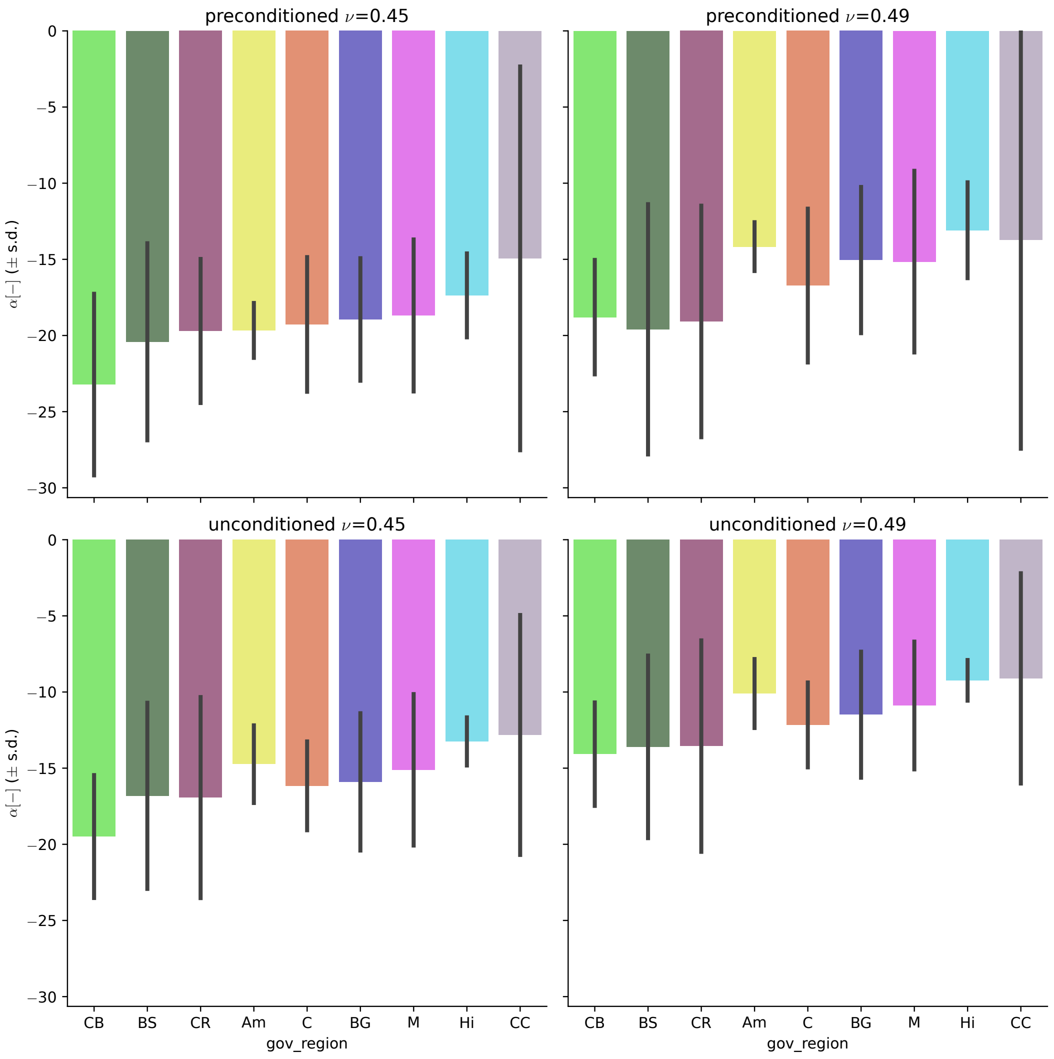

Figure S4: Resulting values for the nonlinearity parameter α, averaged over the governing regions.

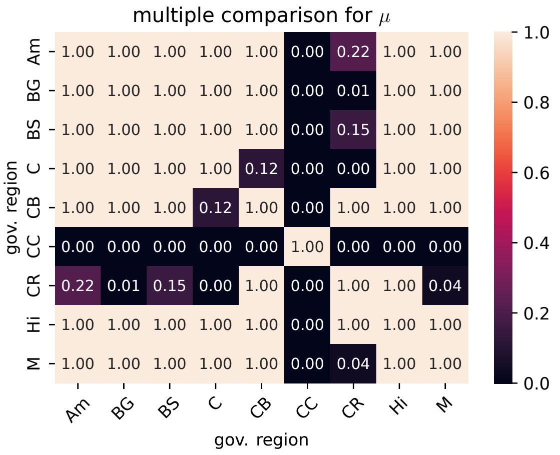

$\nu = 0.45$ and unconditioned data

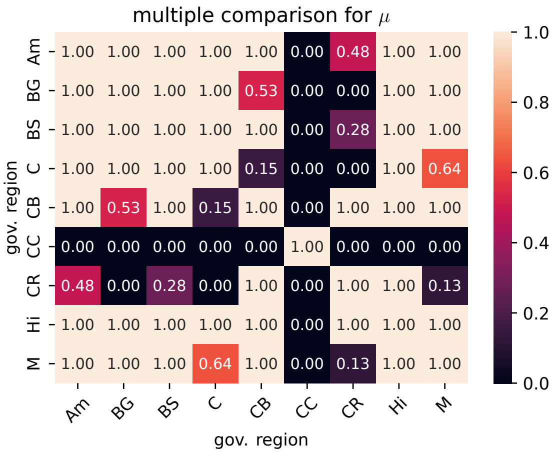

$\nu = 0.45$ and preconditioned data

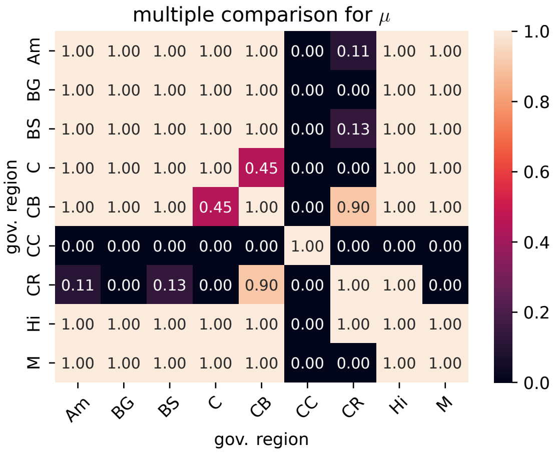

$\nu = 0.49$ and unconditioned data

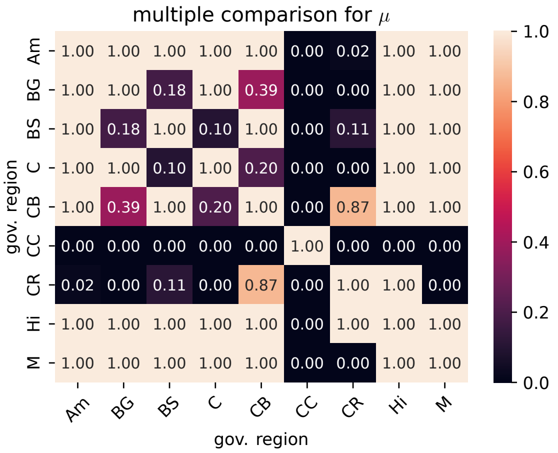

$\nu = 0.49$ and preconditioned data

Figure S5: Resulting p-values from pairwise post hoc Mann-Whitney-U tests comparing the shear modulus μ for the different governing regions. Low p values indicate that the corpus callosum and the corona radiata have distinct properties.

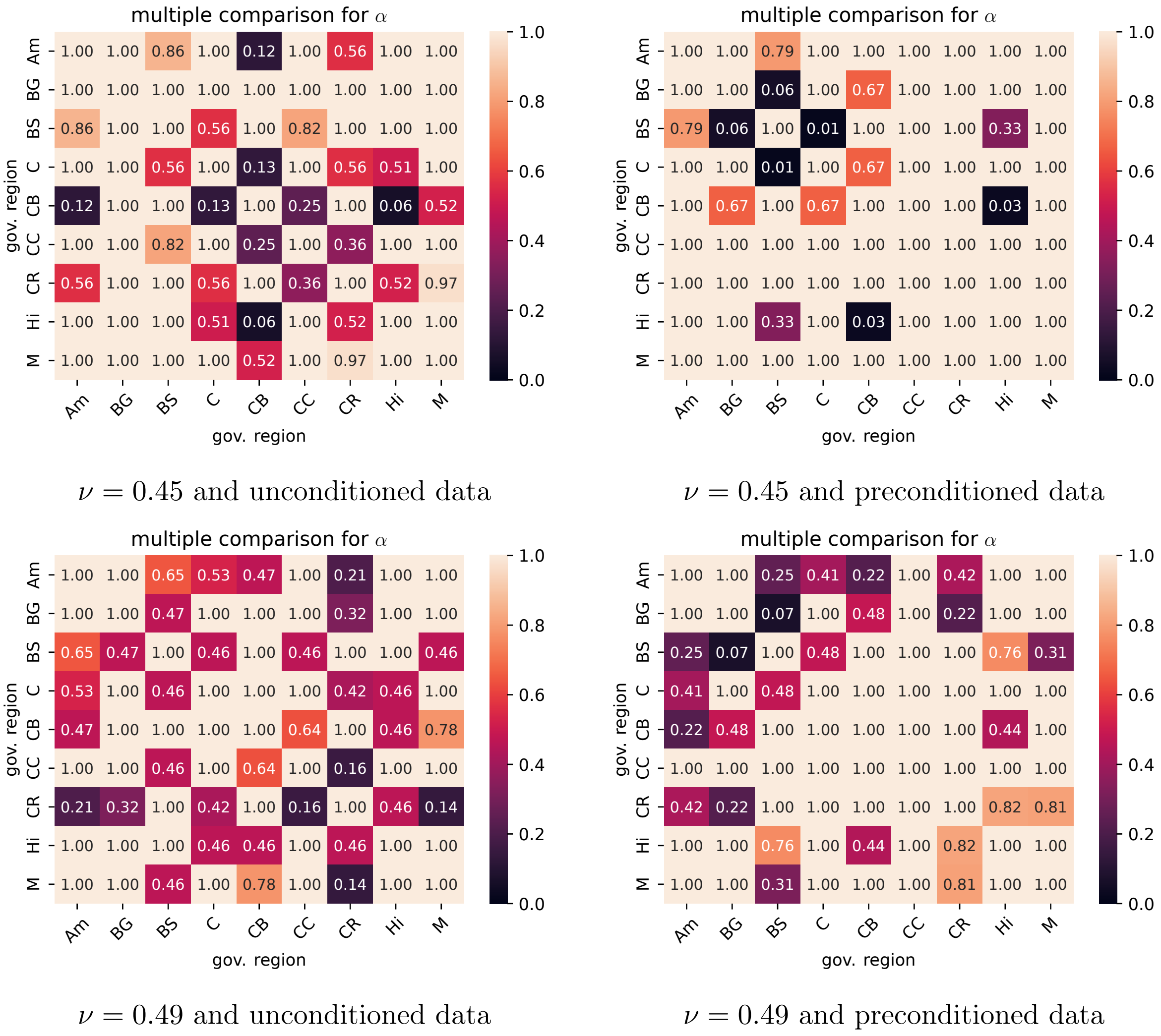

Figure S6: Resulting p-values from pairwise post hoc Mann-Whitney-U tests comparing the nonlinearity parameter α for the different governing regions. A lack of low p values indicates that no distinct regions are found.

4 Inter-individual variation

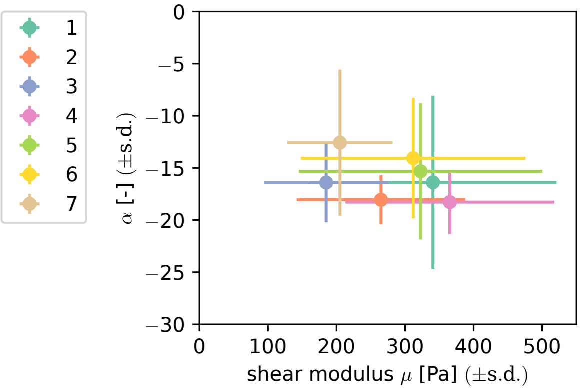

$\nu = 0.45$ and unconditioned data

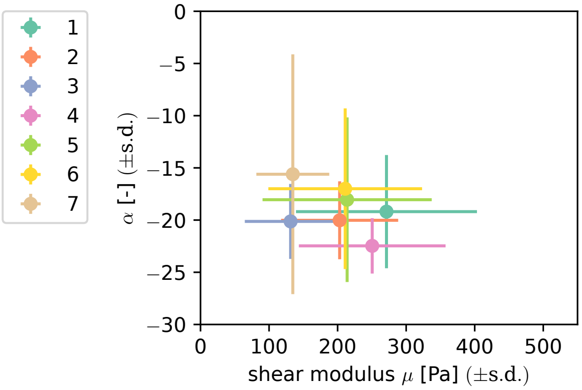

$\nu = 0.45$ and preconditioned data

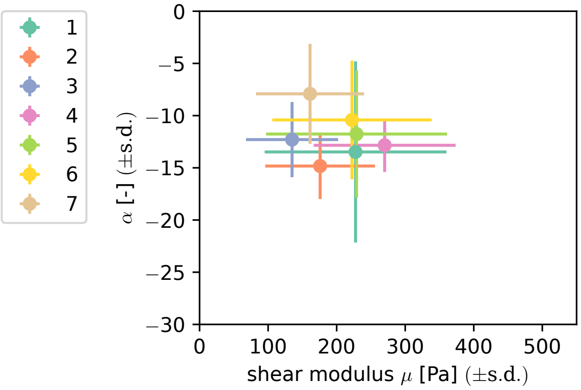

$\nu = 0.49$ and unconditioned data

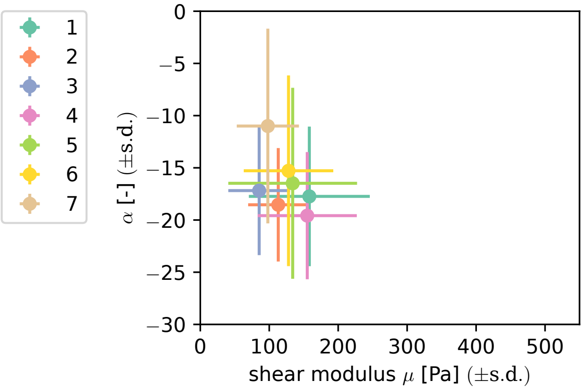

$\nu = 0.49$ and preconditioned data

Figure S7: Parameter values and their standard deviation for different tested brains.

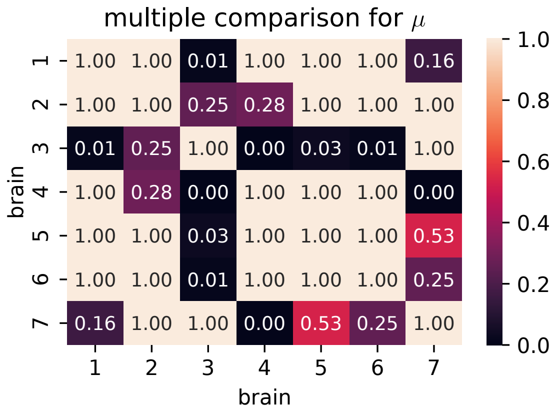

$\nu = 0.45$ and unconditioned data

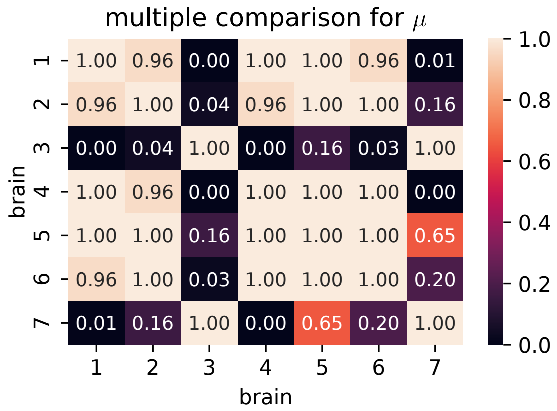

$\nu = 0.45$ and preconditioned data

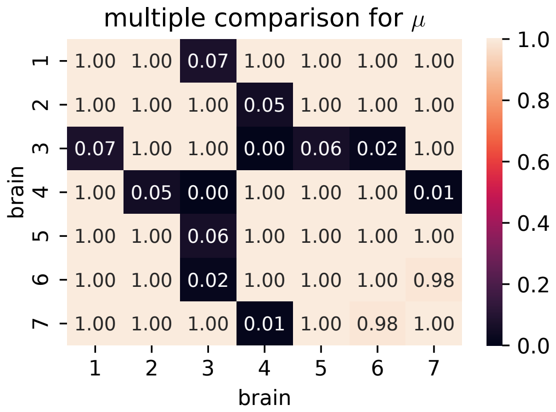

$\nu = 0.49$ and unconditioned data

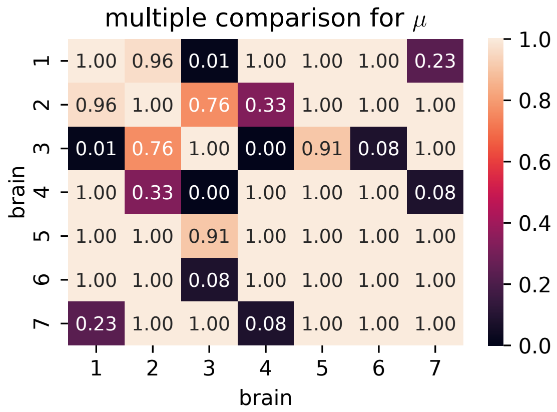

$\nu = 0.49$ and preconditioned data

Figure S8: Resulting p-values from pairwise post hoc Mann-Whitney-U tests comparing the shear modulus μ for different brains. Low p values indicate that the samples from brain 3 show significantly different properties compared to those from the other tested brains.

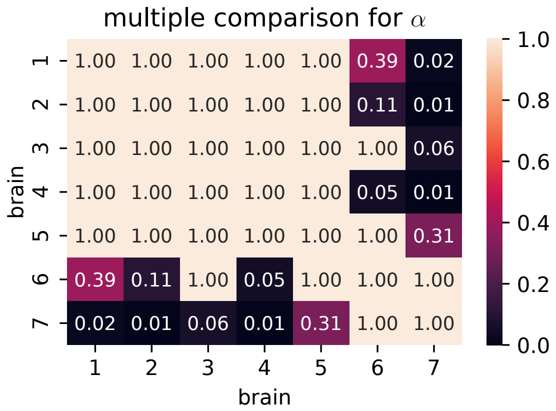

$\nu = 0.45$ and unconditioned data

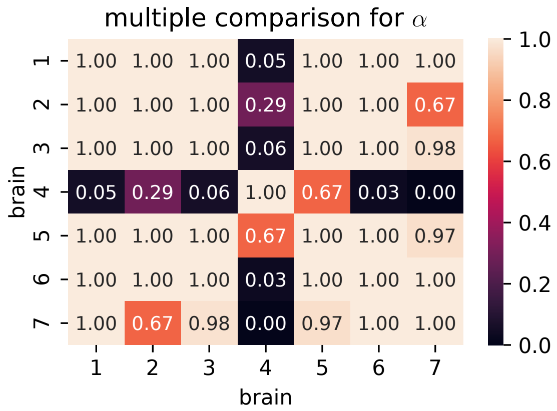

$\nu = 0.45$ and preconditioned data

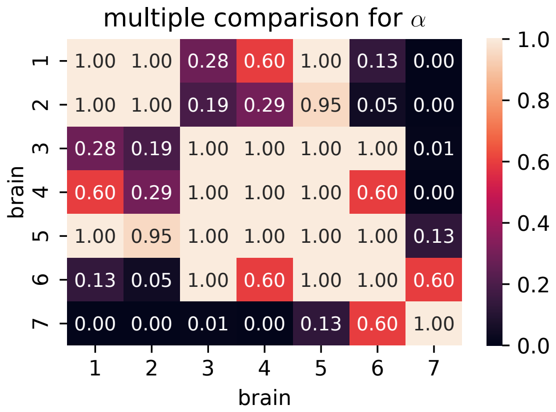

$\nu = 0.49$ and unconditioned data

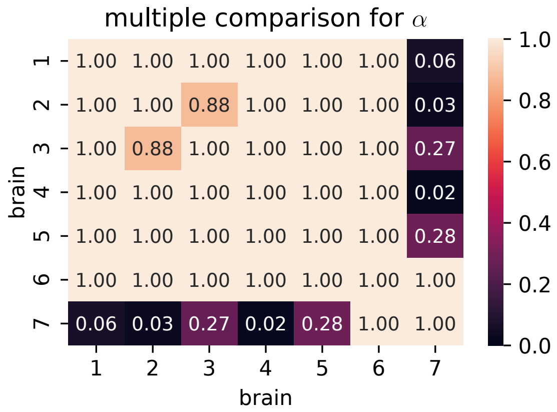

$\nu = 0.49$ and preconditioned data

Figure S9: Resulting p-values from pairwise post hoc Mann-Whitney-U tests comparing the shear modulus μ for different brains. Low p values show significantly different values for brain 7.

5 Influence of compressibility

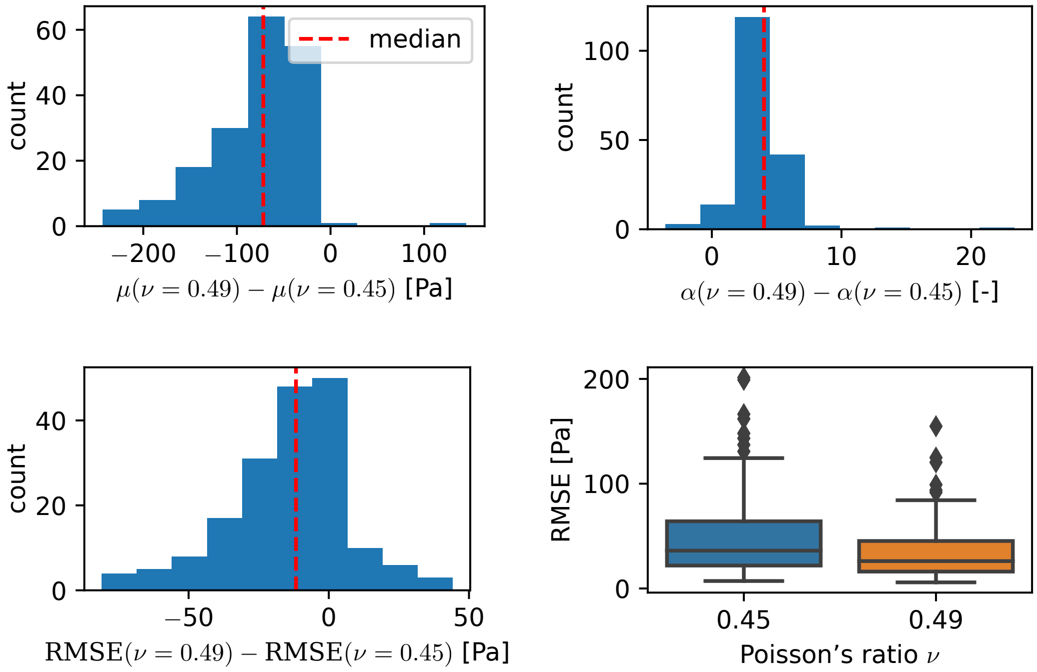

unconditioned data

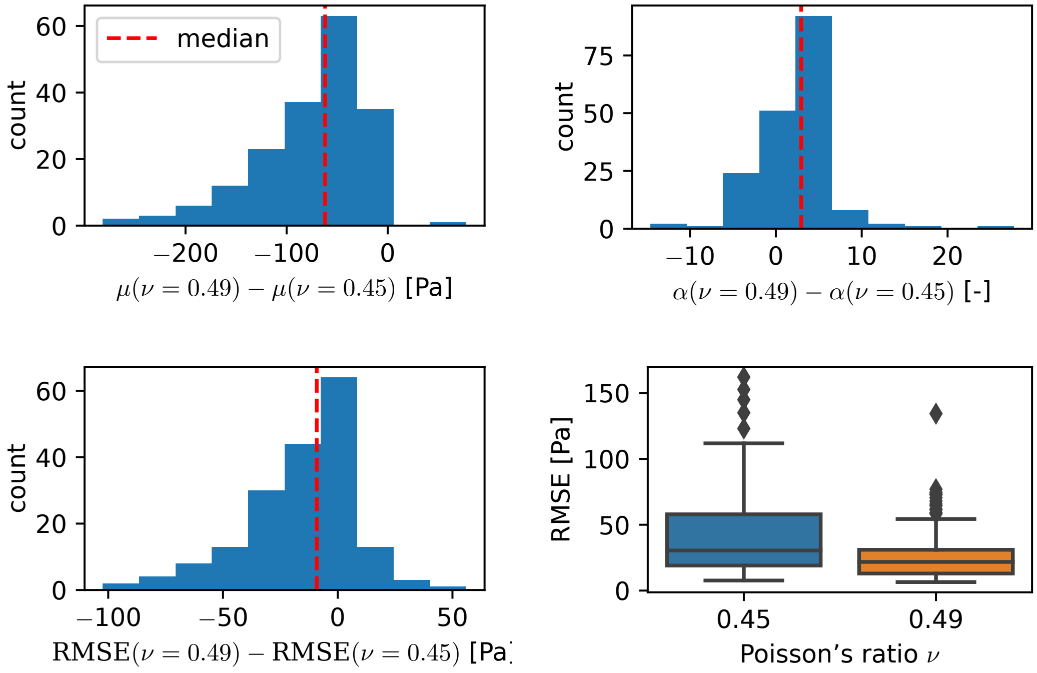

preconditioned data

Figure S10: Pairwise difference for the parameters μ and α and the RMSE between samples fitted with $\nu = 0.45$ and $\nu = 0.49$. The boxplots visualize the distribution of RMSE values to put the calculated differences in relation.

6 Preconditioning behavior

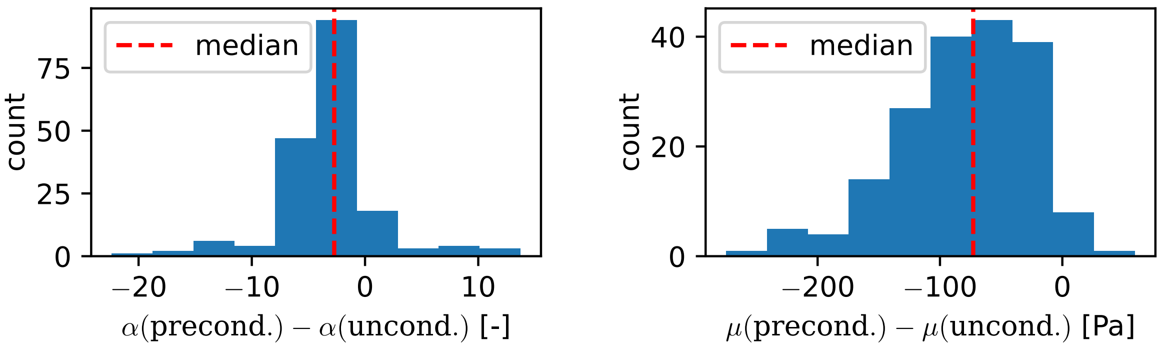

$\nu = 0.45$

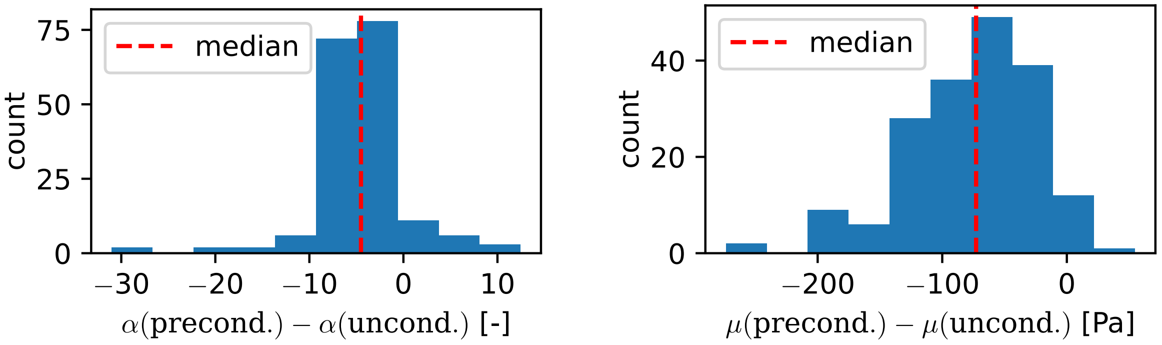

$\nu = 0.49$

Figure S11: Pairwise difference in the material parameters identified through fitting the un- and preconditioned data.

7 Averaging fitted parameters vs. fitting the averaged response

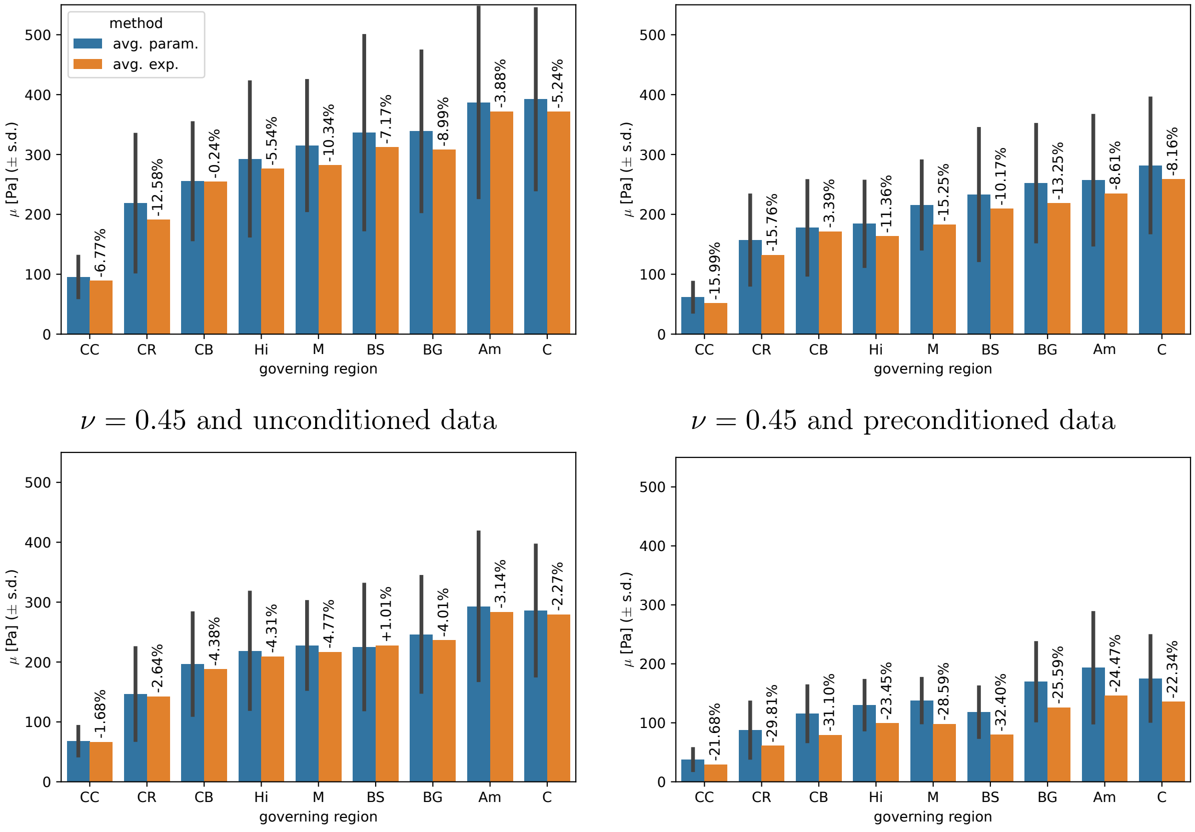

$\nu = 0.45$ and unconditioned data

$\nu = 0.45$ and preconditioned data

$\nu = 0.49$ and unconditioned data

$\nu = 0.49$ and preconditioned data

Figure S12: Comparison between the averaged shear moduli μ obtained from fitting the experimental data of each specimen separately and those obtained from fitting the averaged experimental response.

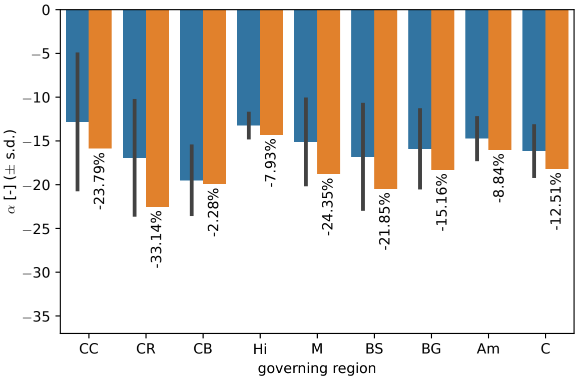

$\nu = 0.45$ and unconditioned data

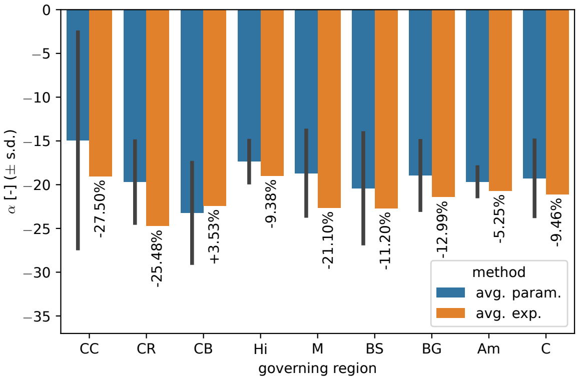

$\nu = 0.45$ and preconditioned data

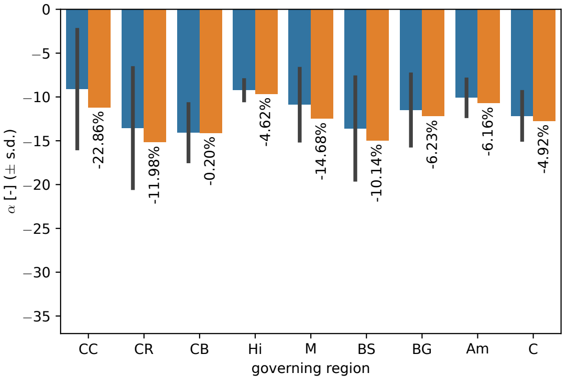

$\nu = 0.49$ and unconditioned data

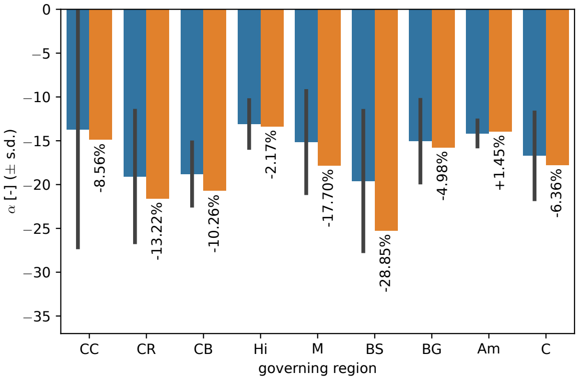

$\nu = 0.49$ and preconditioned data

Figure S13: Comparison between the nonlinearity parameter α obtained from fitting the experimental data of each specimen separately and those obtained from fitting the averaged experimental response.

8 Global optimality

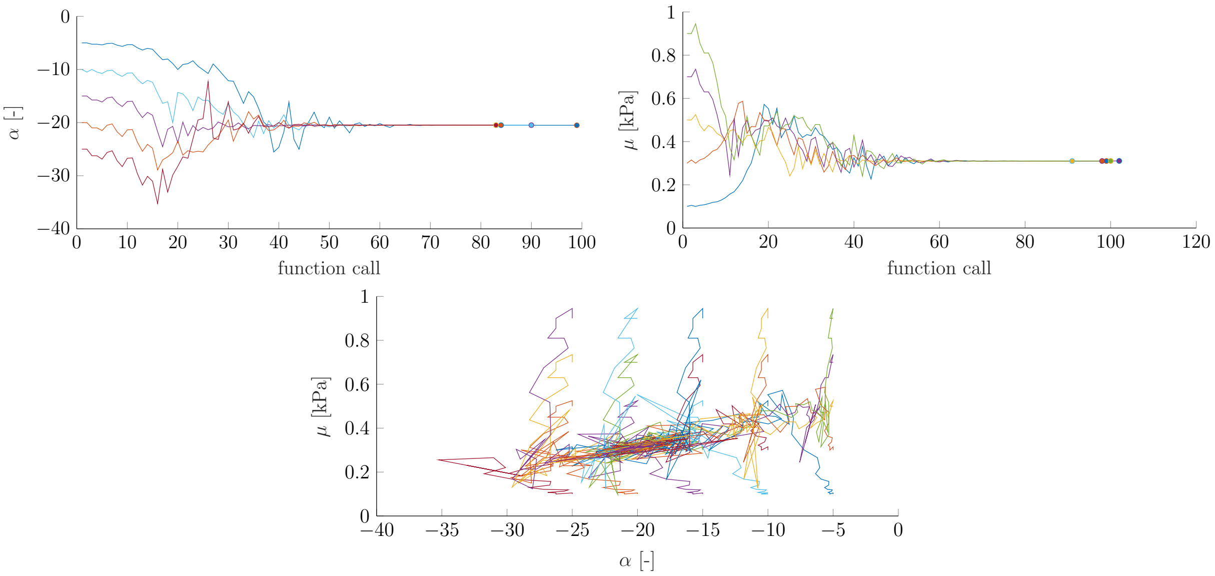

Figure S14: Global optimality study, where the optimization using the Nelder-Mead algorithm was started with different initial parameter values. The parameter values of the converged solution are close together, indicating no problems with local minima.

9 On the generalization capabilities of identified model parameters

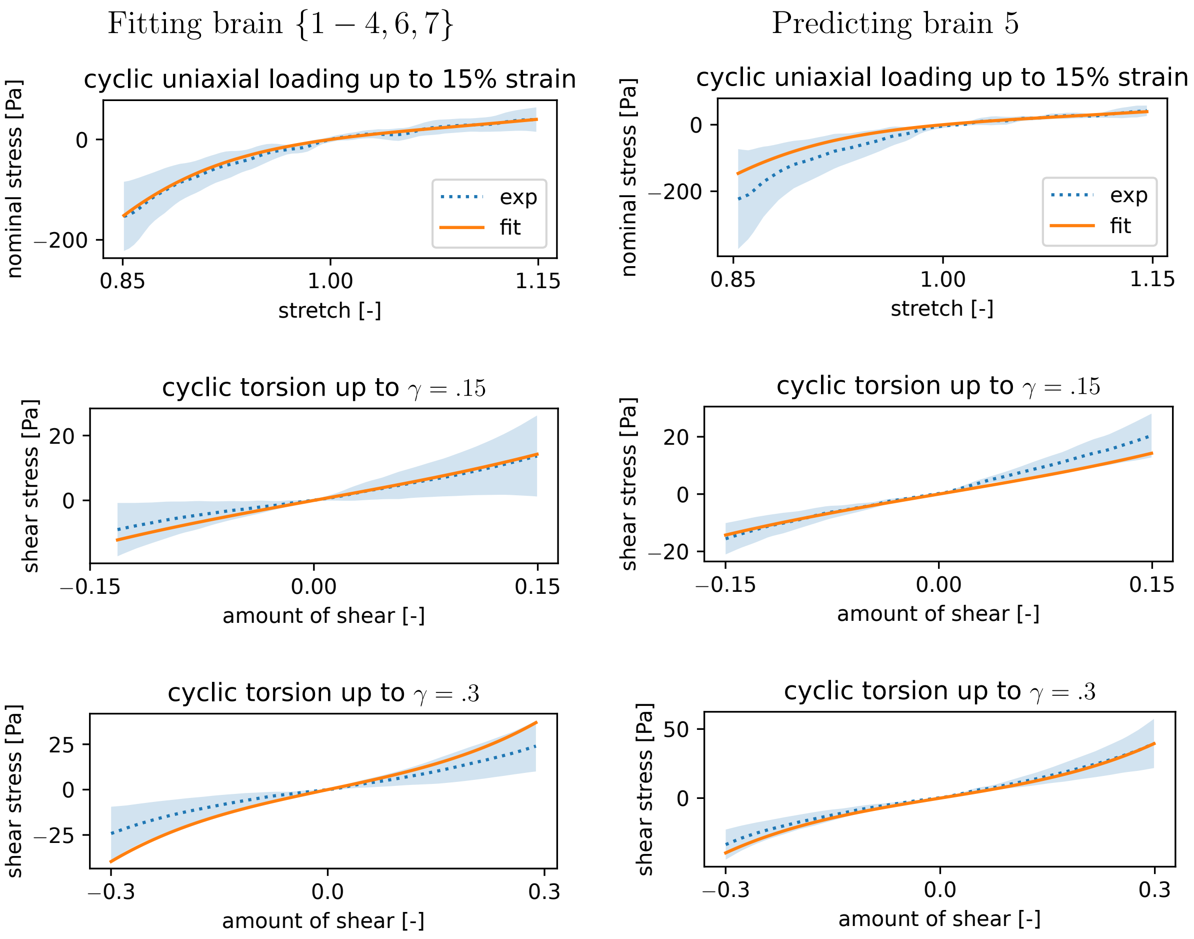

Figure S15: Generalization capabilities of the fitted parameters for the corpus callosum, the first cylce, and a Poisson's ratio of $\nu = 0.45$. First, we identify parameters using the averaged experimental data of the corpus callosum from all brains but number five (left). Subsequently, we use the obtained parameters to simulate the averaged response of the corpus callosum for the remaining brain number five (right). Standard deviations of the experimental data are visualized by the shaded blue areas.

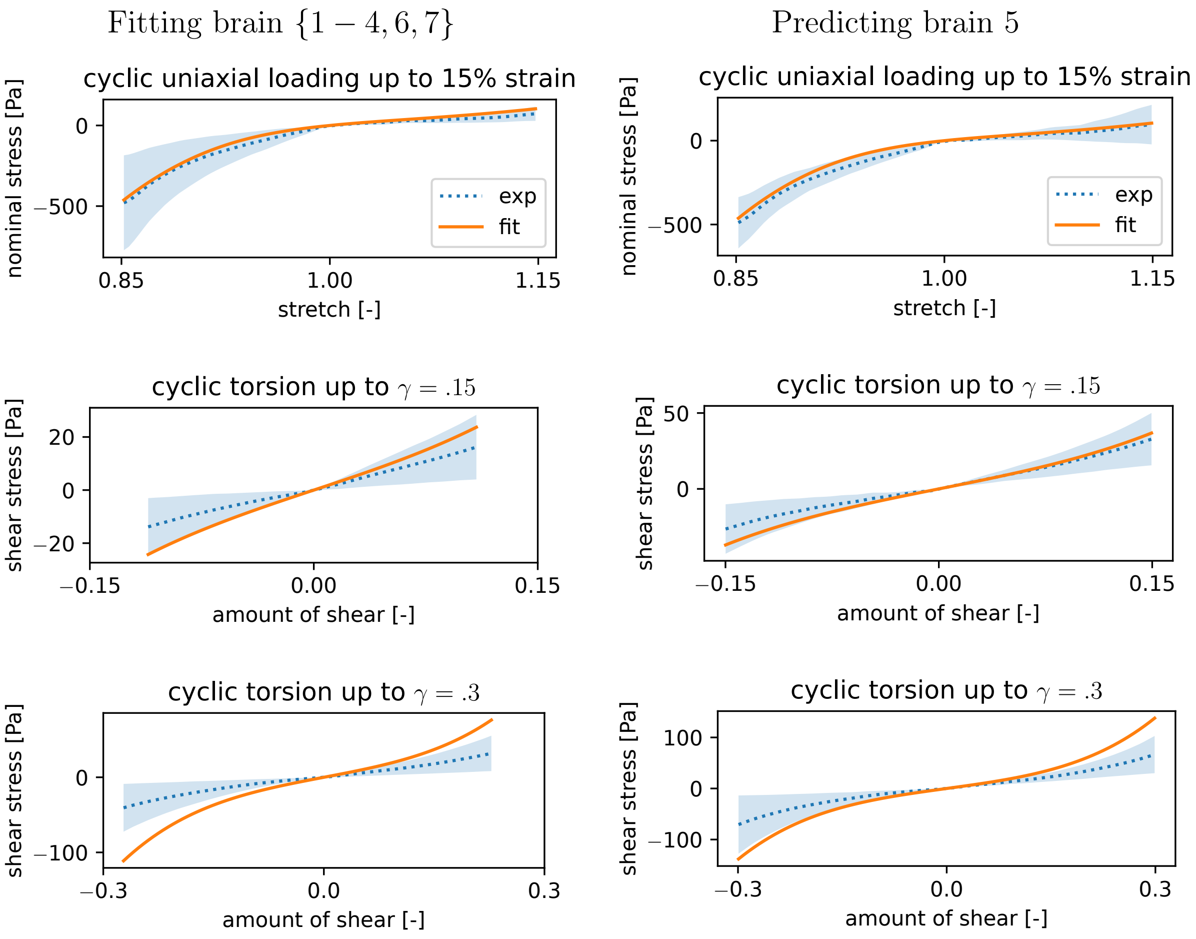

Figure S16: Generalization capabilities of the fitted parameters for the corona radiata, the first cylce, and a Poisson's ratio of $\nu = 0.45$. First, we identify parameters using the averaged experimental data of the corona radiata from all brains but number five (left). Subsequently, we use the obtained parameters to simulate the averaged response of the corona radiata for the remaining brain number five (right). Standard deviations of the experimental data are visualized by the shaded blue areas.
